# An Uncommon Case of Splenic Abscess with Gangrenous Gas Necrosis

**DOI:** 10.1155/2022/9279418

**Published:** 2022-01-15

**Authors:** Sunil Basukala, Bishnu Deep Pathak, Gaurab Mainali, Bikash Bahadur Rayamajhi, Raveesh Mishra, Narayan Thapa

**Affiliations:** ^1^Department of Surgery, Nepalese Army Institute of Health Sciences, College of Medicine, Kathmandu, Nepal; ^2^Nepalese Army Institute of Health Sciences, College of Medicine, Kathmandu, Nepal; ^3^Department of Anaesthesiology and Critical Care Medicine, Nepalese Army Institute of Health Sciences, College of Medicine, Kathmandu, Nepal

## Abstract

Splenic abscess is a rare clinical entity with diagnostic challenges. Though rare, it is potentially a life-threatening clinical condition with high mortality reaching more than 70%. The common signs and symptoms include the triad of fever, left upper quadrant tenderness, and leukocytosis. Early diagnosis, however, can readily be made by the combination of clinical features, abdominal ultrasonography (USG), and computed tomography (CT). The management of splenic abscess includes medical therapy, CT-guided percutaneous aspiration, and splenectomy. We, hereby, present a rare case of splenic abscess with gangrenous gas necrosis, who underwent splenectomy.

## 1. Introduction

Splenic abscess is a potentially life-threatening clinical entity with an incidence of 0.14% to 0.70%. In the past, the mortality rate of patients with splenic abscess was near 100% due to its nonspecific presentation and delayed diagnosis [1, 2]. The predisposing factors for splenic abscess include conditions that compromise the immune system, such as endocarditis, diabetes mellitus, congenital or acquired immunodeficiency, and use of immunosuppressants [3]. Other risk factors include trauma and intravenous drug abuse in addicts [4]. The clinical manifestations usually include the left upper abdominal pain, fever, nausea, vomiting, and anorexia in various combinations [5]. The management of splenic abscess is based on medical therapy with antibiotics and surgery or percutaneous drainage [2, 3, 5].

We, hereby, present a rare case of splenic abscess with gangrenous gas necrosis who underwent splenectomy.

## 2. Case Report

A 64-year-old ex-serviceman presented to the Emergency Department (ED) with fever (recorded up to 39.0 °C), persistent left upper quadrant abdominal pain and multiple episodes of vomiting for the last one week. He did not have any complaints of altered bowel and bladder habits. He was a chronic alcoholic with uncontrolled type 2 Diabetes Mellitus under irregular oral medications. There was no history of recent surgeries, hospital admission, or trauma.

**Figure 1:**
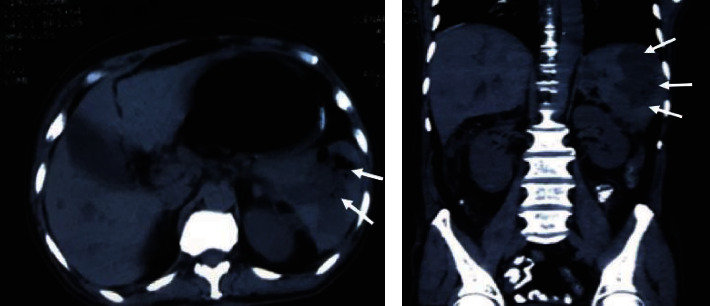
NCCT scan showing the intrasplenic gas-containing collection, consistent with abscess (shown by white arrows).

On physical examination, we noted the presence of a fever of 38.8 °C and pulse rate of 110 beats per minute but normal blood pressure. On abdominal examination, there was tenderness on the left hypochondriac region with mild splenomegaly up to two centimetres below the left subcostal area. His laboratory parameters (Table 1) showed Hemoglobin (Hb)-10.0 g/dL, Erythrocyte Sedimentation Rate (ESR) -32 mm/1^st^ hour, and Total Leucocyte Count (TLC) -12.5 × 10^9^/L with neutrophilia (78%). His Renal Function Test (RFT) showed raised urea and creatinine of 223 and 4.4 mg/dL, respectively. Human Immunodeficiency Virus (HIV), Hepatitis B Surface Antigen (HBsAg), and Hepatitis C Virus (HCV) serology were negative. The stool examination along with the Widal test was normal. Liver Function Test (LFT) showed total bilirubin 2.0 mg/dL, direct bilirubin 1.4 mg/dL, and Alkaline Phosphatase (ALP) -245 U/L. Fluid resuscitation with compound sodium lactate was started in the Emergency Department (ED). After resuscitation with intravenous fluids, broad-spectrum intravenous antibiotics and intravenous analgesics were given. On radiological examination, ultrasonography of the abdomen and pelvis showed echogenic fluid noted in the perisplenic region with no vascularity and low-level echoes floating within it. The patient was admitted with a provisional diagnosis of splenic abscess with impending Acute Kidney Injury (AKI). He underwent hemodialysis two episodes the following day for AKI. Due to the raised serum creatinine level, the patient was planned for Noncontrast Computed Tomography (NCCT) the following day, which showed mild splenomegaly with large ill-defined fluid attenuating lesion measuring about 10.6 × 9.2 × 8.0 cm seen in spleen extending to splenic hilum, suggestive of splenic abscess (Figure 1).

We tried percutaneous aspiration of the splenic abscess, but the attempt was unsuccessful. Despite aggressive supportive care and hemodialysis, the patient continued to have persistently worsening thrombocytopenia, anemia, renal failure, and metabolic acidosis with respiratory compensation (Table 1). His clinical status continued to decline. The patient was planned for laparotomy with a midline incision the following day, 48 hours after the admission in the hospital.

**Figure 2:**
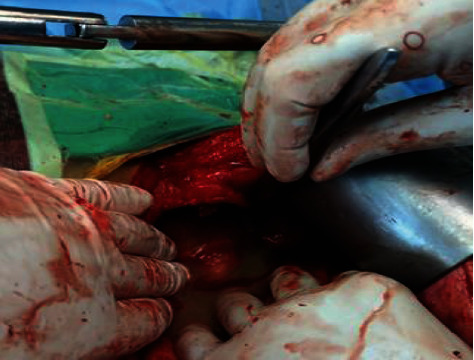
A localized collection of pus in the lesser sac of the abdomen.

**Figure 3:**
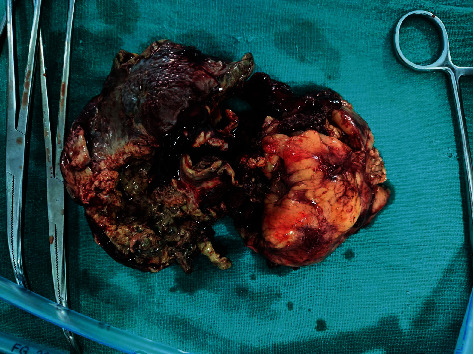
Gross specimen of the gangrenous spleen after splenectomy.

Intraoperatively, the spleen was exposed, allowing visualization of a voluminous perforated abscess in the lesser sac (Figure 2), which revealed a ruptured splenic abscess. The short vessels were sectioned using a sealing device; the main vessels were controlled using nonabsorbable ligatures, staying at distance from the pancreas; and splenectomy was done (Figure 3). After local washing, a drain was left in place and the abdominal wall was closed using absorbable stitches. The intra-abdominal fluid sample came out to be positive for *Clostridium perfringens*. The diagnosis of splenic abscess was confirmed by histopathological examination.

Postoperatively, the patient was transfused three pints of whole blood and was managed in the Intensive Care Unit (ICU). He underwent regular hemodialysis in ICU. His AKI gradually improved. After that, he was shifted to the ward. His postoperative stay was uneventful and was discharged ten days after his stay in the ward.

## 3. Discussion

A splenic abscess usually occurs in a patient with an underlying disorder, including infection, emboli, trauma, recent surgery, malignant hematologic conditions, and immunosuppression. Nowadays, lifestyle changes have resulted in increasing prevalence of Diabetes Mellitus, malignancies, and immunosuppression. These predisposing factors increase the risk of development of a splenic abscess. In a retrospective review of 67 cases of splenic abscess done by Chang et al., 54 of them had an underlying predisposing disease. Out of these, Diabetes Mellitus was the leading cause [6]. It was also supported by another retrospective review of 75 cases of splenic abscess by Sreekar et al. [7]. Our patient was a chronic alcoholic and had uncontrolled type 2 Diabetes Mellitus.

Gram-negative bacilliare predominantly seen in most of the cases and common organisms grown are *Klebsiella pneumonia*, *Escherichia coli*, and *Staphylococcus aureus* [8]. In contrast to it, our patient showed positive for *Clostridium perfringens* in the intra-abdominal fluid sample. Chalasani et al. also reported a case of splenic abscess due to *Clostridium perfringens* [9].

The clinical signs and symptoms of splenic abscess are non-specific. The patients may present with a triad of fever, left upper quadrant pain, and palpable tender abdominal mass [2]. Our case also had similar clinical findings. Fever, persistent abdominal pain on the left upper quadrant, and tender mass were present in our case. Splenic abscesses may often be misdiagnosed because of their vague signs and symptoms. Modern imaging has aided for proper diagnosis. Ultrasonography and CT scan of the abdomen are the diagnostic modalities [10].

Intravenous antimicrobial therapy, CT-guided percutaneous aspiration, and splenectomy are different treatment options for splenic abscess [8]. The best treatment options remain unclear. Percutaneous drainage should be performed when there is a unilocular abscess. The splenectomy is preferred for multiloculated abscess [11]. The studies have shown that percutaneous drainage may be appropriate initially, but there is a high failure rate. Therefore, surgery remains the standard treatment [12]. But, according to a study done by Schweh et al., out of 42 patients who had undergone percutaneous drainage, only two patients had unsuccessful results [13]. Our patient also had unsuccessful CT-guided percutaneous aspiration and was finally managed with successful splenectomy.

The most common complications of splenic abscess are hemorrhage, pneumothorax, pleural effusion, and colonic injury [8]. However, no intra- and postoperative complications were seen in our patient. Studies have shown that the mean length of hospital stay of patients who underwent surgery was 15.83 days [7]. Our patient was discharged after the 13^th^ postoperative day.

## 4. Conclusion

Splenic abscess is a rare entity presenting with vague signs and symptoms. It is a life-threatening condition with high morbidity and mortality. So, any febrile patients with the left upper quadrant tenderness and leukocytosis should be suspected of splenic abscess, and diagnosis must be confirmed by imaging studies.

## Figures and Tables

**Table 1:** Laboratory parameters on admission and during 1^st^ surgical intervention.

s.no	Laboratory test	Normal range	On admission	During laparotomy	Postoperative
1.	WBC count (cells/L)	4.5–11.0 × 10^9^	12.5 × 10^9^	21 × 10^9^	16 × 10^9^
2.	Neutrophil (%)	50–70%	73	79	87
3.	Lymphocyte (%)	20–40%	18	17	12
4.	Hemoglobin (g/dL)	12–16 g/dL	10.0 g/dL	9.3 g/dL	11.1 g/dL
5.	Platelet count (10^9^ cells/L)	125–350 × 10^9^	180	170	210
6.	Hematocrit (%)	36−48%	42	44	41
7.	Total bilirubin	0.1–1.2 mg/dL	2.0	2.8	2.6
8.	Direct bilirubin	<0.3 mg/dL	1.4	1.9	2.1
9.	AST (U/L)	5–45 U/L	35	127	149
10.	ALT (U/L)	5–40 U/L	26	111	102
11.	ALP (U/L)	44–147 U/L	245	289	223
12.	Albumin (g/L)	3.5–5.5 mg/L	3.7	3.4	3.3
13.	Blood sodium level (mEq/L)	135–145 mEq/L	139	137	145
14.	Blood potassium level (mEq/L)	3.6–5.2 mEq/L	3.9	3.7	4.7
15.	Blood urea nitrogen (mg/dL)	8–20 mg/dL	223	110	120
16.	Creatinine (mg/dL)	0.5–1.2 mg/dL	4.4	1.2	3.4

## Data Availability

We do not have additional data or supplementary files. All the required information have been included in the manuscript itself.
